# FLYNC: a machine-learning-driven framework for discovering long noncoding RNAs in *Drosophila melanogaster*

**DOI:** 10.1093/nargab/lqaf216

**Published:** 2026-01-15

**Authors:** Ricardo F dos Santos, Tiago Baptista, Graça S Marques, Catarina C F Homem

**Affiliations:** iNOVA4Health, NOVA Medical School, Faculdade de Ciências Médicas, NMS, FCM, Universidade Nova de Lisboa, Lisbon, 1150-082, Portugal; iNOVA4Health, NOVA Medical School, Faculdade de Ciências Médicas, NMS, FCM, Universidade Nova de Lisboa, Lisbon, 1150-082, Portugal; iNOVA4Health, NOVA Medical School, Faculdade de Ciências Médicas, NMS, FCM, Universidade Nova de Lisboa, Lisbon, 1150-082, Portugal; iNOVA4Health, NOVA Medical School, Faculdade de Ciências Médicas, NMS, FCM, Universidade Nova de Lisboa, Lisbon, 1150-082, Portugal

## Abstract

Noncoding RNAs have increasingly recognized roles in critical molecular mechanisms of disease. However, the noncoding genome of *Drosophila melanogaster*, one of the most powerful disease model organisms, has been understudied. Here, we present FLYNC—FLY noncoding RNA discovery and classification—a novel explainable boosting machine model that accurately predicts the probability of a newly identified RNA transcript being a long noncoding RNA (lncRNA). Integrated into an end-to-end bioinformatics pipeline capable of processing single cell or bulk RNA sequencing data, FLYNC outputs potential new noncoding RNA genes. FLYNC leverages large-scale genomic and transcriptomic datasets to identify patterns and features that distinguish noncoding genes from protein-coding genes, thereby facilitating lncRNA prediction. We demonstrate the application of FLYNC to publicly available *Drosophila* adult head bulk transcriptome and single-cell transcriptomic data from *Drosophila* neural stem cell lineages and identify several novel tissue- and cell-specific lncRNAs. We have further experimentally validated the existence of a set of FLYNC predicted lncRNAs by RT-PCR and RNA PolII binding. Overall, our findings demonstrate that FLYNC serves as a robust tool for identifying lncRNAs in *D. melanogaster*, transcending current limitations in ncRNA identification and harnessing the potential of machine learning.

## Introduction

Traditionally, the study of stem cell function during animal development or tissue maintenance has been primarily focused on the protein-coding genome. However, recent transcriptomic analyses revealed that coding genes constitute a small fraction of the genome, while a significantly larger portion of the genome is transcribed [1]. This extensive transcription of noncoding genome regions has been observed across various organisms, ranging from *Drosophila* to human [2, 3].

Despite the wealth of knowledge derived from studying protein-coding genes (PCGs), research on noncoding genome has however lagged [4], likely due to the technical challenges associated with identifying and characterizing the function of noncoding RNAs (ncRNAs) *in vivo*. Noncoding regions encompass a diverse array of transcripts, with the most prevalent being long noncoding RNAs (lncRNAs). lncRNAs are defined as RNAs longer than 200 nucleotides that do not encode functional proteins [5]. lncRNAs have been identified as regulators of various cellular processes, including chromatin dynamics, transcriptional regulation, translation control, pluripotency, and differentiation [6].

Interestingly, a large fraction of lncRNAs were found to be expressed specifically in the brain, in a cell type and developmental stage-specific manner, correlating with the complex requirement for spatiotemporal gene regulation during brain development and neural differentiation [7]. Nevertheless, the exact *in vivo* functions of lncRNAs remain unclear since their molecular functions have primarily been explored *in vitro* [8–14].

In fact, the study of individual lncRNAs poses significant challenges in vertebrates due to the intricate and time-consuming process of mutant generation. In contrast, *Drosophila melanogaster* emerges as an excellent model system for *in vivo* studies elucidating the functional roles of lncRNAs. Its genetic tractability, short generation time, well-characterized and conserved developmental processes and well-characterized cell lineages collectively render it an ideal platform for investigating complex biological phenomena. However, despite these advantages, the noncoding genome of *D. melanogaster* remains poorly characterized, hindering the systematic utilization of this model for studying the *in vivo* function of lncRNAs. Therefore, there is an urgent need for a comprehensive characterization of *Drosophila*’s noncoding genome and identification of lncRNAs. Such efforts will facilitate the systematic and integrated study of lncRNA function *in vivo* during animal development.

As lncRNAs exhibit weak conservation at the genomic sequence level, relying solely on genomic sequence for their identification poses significant challenges [15]. Also, considering the limited understanding of the sequence–structure–function relationship of lncRNAs, their identification has primarily relied on transcriptome studies, namely via the advent of high-throughput RNA-sequencing technology [16, 17]. However, lncRNA cell specificity combined with the relatively low expression levels of most lncRNAs pose challenges in their detection through bulk transcriptomic methods, particularly in weakly sampled datasets [18]. These challenges can now be addressed with the analysis of single-cell transcriptomic datasets. With the emergence of platforms like 10× Genomics, numerous *Drosophila* single-cell datasets are available for analysis, spanning various tissues, organs, developmental stages, and mutant backgrounds [19, 20]. These datasets offer unprecedented opportunities for precise identification of cell-specific noncoding genes (NCGs) in *Drosophila*, enabling comprehensive investigations into the regulatory roles and functional significance of lncRNAs throughout development and in diverse biological contexts.

However, accurate automated or manual annotation of lncRNA genes remains a challenging endeavor. Automated annotation relies on transcriptome assembly approaches, which are efficient and cost-effective but often yield incomplete and inaccurate annotations, leading to numerous false positives. On the other hand, manual annotation, exemplified by databases like RefSeq or GENCODE, involves rigorous experimental validation resulting in high-quality data, while being resource-intensive and time-consuming [16].

Machine-learning-based approaches have shown great promise in addressing various biological questions [21]. These approaches can leverage large-scale genomic and transcriptomic data to identify patterns and features that distinguish ncRNAs from PCGs, thereby facilitating the prediction of functional ncRNAs in newly identified RNA transcripts [22]. The integration of machine learning (ML) techniques with biological data has the potential to uncover novel ncRNAs and contribute to a more comprehensive understanding of the noncoding genome.

Existing models for lncRNA prediction have largely been developed using mammalian data and some provide broad cross-species application [23, 24]. While such frameworks can be retrained for *Drosophila*, they are not optimized for the distinct genomic architecture and epigenomic landscape of this species, which limits their predictive accuracy. To the best of our knowledge, no model specifically tailored for *D. melanogaster* has been available to date.

Here, we present FLYNC (FLY noncoding RNA discovery and classification), a novel ML model designed to accurately predict whether newly identified RNA transcripts in *D. melanogaster* are noncoding or protein-coding. FLYNC employs an explainable boosting machine (EBM) model, a highly interpretable, glass-box model that combine the predictive power of gradient boosting with full transparency. It integrates multiple layers of information—genomic and epigenomic signals (e.g. GC content, ChIP-seq, RNA Pol II binding, histone marks, phylogenetic conservation), transcript-level annotations [transcription factor (TF) binding profiles], and sequence-based features (*k*-mer frequencies, RNA secondary structure). By leveraging *Drosophila*-specific training data and regulatory features, FLYNC captures species-specific signatures that broadly trained models cannot, resulting in improved sensitivity and specificity in lncRNA prediction.

Beyond its predictive capacity, FLYNC forms an integral part of an end-to-end bioinformatics pipeline that processes sequencing data and generates potential new ncRNA genes, providing an essential resource for annotating the *Drosophila* noncoding genome. This framework represents a step forward for ncRNA research in *Drosophila*, enabling more accurate identification of lncRNAs and facilitating studies on their functional roles in development and broader biological contexts.

## Materials and methods

### Data sources

For the development of FLYNC, we integrated data from three reputable and widely recognized sources, each serving a distinct purpose and contributing unique datasets necessary for the comprehensive analysis of *D. melanogaster* genomics data:

#### Sequence read archive

The Sequence Read Archive (SRA; ncbi.nlm.nih.gov/sra) was selected as the primary source for sequencing reads due to its extensive repository of high-throughput sequencing data. The SRA is managed by the National Center for Biotechnology Information and is part of the International Nucleotide Sequence Database Collaboration. It provides a platform for the storage and access of sequence data from a multitude of species and is instrumental in ensuring that the sequencing data used in our pipeline are both current and comprehensive. By leveraging the SRA’s remote access capabilities, our pipeline can directly download specific datasets on demand, ensuring efficient data management and up-to-date information for analysis.

#### Ensembl

Ensembl (ensembl.org) was chosen for sourcing reference genome annotations of *D. melanogaster*. The *D. melanogaster* reference genome used in this study was release BDGP6.46.113 r103. Ensembl is a project that provides a centralized resource for researchers seeking genome annotations. It is known for its high-quality, manually curated annotation data. The use of Ensembl annotations in our pipeline allows for the precise identification of genomic elements, such as genes, exons, and regulatory regions.

#### University of California, Santa Cruz Genome Browser

The University of California, Santa Cruz (UCSC) Genome Browser (genome.ucsc.edu) was utilized to extract genome features necessary for the construction of our ML model. The UCSC Genome Browser is a sophisticated and interactive tool that provides a user-friendly interface for visualizing genomic data and extracting features such as chromatin states, TF binding sites, expression profiles, and comparative genomics information. The integration of UCSC data into our pipeline provides a rich feature set that enhances the FLYNC ML classifier’s ability to identify patterns and make predictions based on the genomic context. The comprehensive data available through UCSC are invaluable for the development of a robust and predictive bioinformatics pipeline.

### Computational resources

#### Workstation specification

For the development and testing of FLYNC we used Intel^®^ Core™ i7-9700 computer with 64GB DRAM and NVIDIA Quadro P2000 GPU with 5GB GDDR5. This workstation has been used throughout the development of FLYNC and SUBCELL.

#### HPC information and acknowledgements

The HPC service from the Lisbon node (cirrus.a.incd.pt) of Infrasturutra Nacional de Computação Distribuída (INCD) running CentOS 7 was mainly used for compute- and/or memory-intensive tasks, such as read alignment, transcriptome assembly, ML model training and evaluation.

#### Training dataset and ML

To explore our evidence-based approach for distinguishing lncRNAs from PCGs in *D. melanogaster*, we initially prepared a pretraining sample dataset comprising 10 000 randomly selected genes with balanced representation of NCGs and PCGs.This sample dataset facilitated rapid assessment of suitable ML algorithms using the lazypredict Python package, which provides automated model comparison across multiple algorithms with consistent evaluation metrics (Supplementary Table S1).

Based on the initial algorithm screening, tree-based algorithms performed consistently well on the pretraining dataset. We selected the EBM from Microsoft’s “interpret” library (https://www.semanticscholar.org/paper/InterpretML%3A-A-Unified-Framework-for-Machine-Nori-Jenkins/60baa46784e8e9a30a57e1875907d008fbdc817b) as our final classification algorithm. This decision was informed by EBM tree-based implementation’s performance capabilities and its inherent interpretability advantages for biological applications.

Our ML pipeline integrated multiple frameworks to optimize different aspects of the workflow: (i) “scikit-learn” for data preprocessing and model evaluation: scikit-learn, (ii) “interpret” library for EBM implementation, (iii) Custom scripts interfacing with UCSC Genome Browser information repository for feature extraction and engineering. This choice encompasses several reasons: (i) Leveraging Python’s comprehensive data science ecosystem for seamless workflow integration, (ii) Supervised learning focus aligned with our binary classification objective (lncRNA versus PCG prediction), (iii) EBM’s transparent decision-making process facilitating biological interpretation, (iv) Extensive documentation and active development communities ensure long-term sustainability, (v) Consistent APIs and version control enable reproducible research practices.

Detailed descriptions of these features and the rationale behind their selection are discussed in the “Results and discussion” section. The training dataset itself was crafted to incorporate features that are biologically relevant for the discrimination between noncoding and coding RNA transcripts. This carefully selected combination of features was chosen to reflect the functional importance and coding potential of RNA transcripts.

### Evaluation of ML classifier

To objectively assess the performance of the EBM model used in FLYNC, we employed a comprehensive evaluation strategy incorporating multiple validation approaches and addressing class imbalance challenges inherent in the dataset. These metrics provide a comprehensive picture of the model’s predictive accuracy, its ability to distinguish between classes, and the balance between sensitivity and specificity.

#### Data partitioning strategy

The complete annotated dataset was partitioned using a stratified three-way split to ensure representative distribution of both coding and ncRNA classes across all subsets. The data were divided into training (70%), test (15%), and validation (15%) sets. This partitioning strategy was designed to support rigorous model development while maintaining independent datasets for hyperparameter optimization and final performance evaluation. Stratified sampling was employed to preserve the class distribution across all splits, which is particularly critical given the substantial class imbalance in the dataset. The complete training dataset is provided in the Supplementary material (Supplementary Table S2) and through FLYNC’s code repository.

#### Hyperparameter optimization

Hyperparameter optimization was conducted using Optuna [25], a state-of-the-art hyperparameter optimization framework. The training set (70% of data) was used to train candidate models, while the test set (15% of data) served as the evaluation dataset during the optimization trials. This separation ensured that hyperparameter selection was guided by performance on data not seen during training. The optimization objective was explicitly set to maximize precision, reflecting the strategic decision to prioritize the reduction of false positives in lncRNA prediction. The hyperparameter search space encompassed EBM-specific parameters including learning rate, maximum number of boosting rounds, interaction depth, minimum samples per leaf, and binning strategy.

To facilitate reproducibility and enable future model development, FLYNC incorporates a dedicated optimization module with an embedded hyperparameter search space capable of deploying multiple batch studies. The complete Optuna database generated during our experiments, containing the full history of trials and optimized configurations, is provided in the FLYNC code repository, enabling researchers to examine the optimization trajectory and replicate or extend the hyperparameter tuning process.

#### Model evaluation with held-out validation set

Following hyperparameter optimization, the model configuration was retrained using the combined training and test sets (85% of data) to maximize the information available for learning complex patterns distinguishing lncRNAs from PCGs. This retrained model was then evaluated on the validation set (15% of data), which had been held out from all previous training and optimization steps, providing an unbiased estimate of real-world performance.

#### Accuracy

Accuracy is the most intuitive performance measure, and it is simply a ratio of correctly predicted observation to the total observations. It is a useful metric only when the classes are balanced.


\begin{eqnarray*}
{\mathrm{ Accuracy}} = \ \frac{{\mathrm{ TP + TN}}}{{\mathrm{ TP + TN + FP + FN}}},
\end{eqnarray*}

where: TP = true positives; TN = true negatives; FP = false positives; FN = false negatives.

#### Precision (positive predictive value)

Precision is the ratio of correctly predicted positive observations to the total predicted positive observations. High precision relates to the low false positive rate (FPR).


\begin{eqnarray*}
{\mathrm{ Precision}} = \ \frac{{\mathrm{ TP}}}{{\mathrm{ TP + FP}}}.
\end{eqnarray*}

#### Recall (sensitivity, true positive rate)

Recall is the ratio of correctly predicted positive observations to all observations in the actual class. It is also known as sensitivity or the true positive rate (TPR).


\begin{eqnarray*}
{\mathrm{ Recall}} = \ \frac{{\mathrm{ TP}}}{{\mathrm{ TP + FN}}}.
\end{eqnarray*}

#### F1 score

The F1 Score is the weighted average of precision and recall. Therefore, this score takes both false positives and false negatives into account.


\begin{eqnarray*}
{ \mathrm{ F1}\ {\mathrm{ score}} = 2\ \times \ \frac{{\left( {{\mathrm{ Precision}}\ \times {\mathrm{ Recall}}} \right)}}{{\left( {{\mathrm{ Precision}} + {\mathrm{ Recall}}} \right)}}}.
\end{eqnarray*}

#### Confusion matrix

A confusion matrix is a table that is often used to describe the performance of a classification model on a set of test data for which the true values are known. It allows the visualization of the performance of an algorithm.

#### Precision-recall curve

The precision-recall curve shows the trade-off between precision and recall for different thresholds. A high area under the curve represents both high recall and high precision.

#### Receiver operating characteristic curve

The ROC curve is a graphical plot that illustrates the diagnostic ability of a binary classifier system as its discrimination threshold is varied. It is created by plotting the TPR against the FPR at various threshold settings.


\begin{eqnarray*}
\mathrm{ TPR }= \ {\mathrm{ Recall}},
\end{eqnarray*}

\begin{eqnarray*}
\mathrm{ FPR }= \ \frac{{\mathrm{ FP}}}{{\mathrm{ FP + TN}}},
\end{eqnarray*}

where: TPR = True Positive Rate; FPR = False Positive Rate

#### Cross-validation

To validate the robustness and generalization capacity of the final model, we performed stratified five-fold cross-validation using the complete dataset (training + test + validation sets combined). In this procedure, the dataset was partitioned into five equal-sized folds, with stratification ensuring proportional representation of both classes in each fold.

The cross-validation process proceeded iteratively: in each of five iterations, 80% of data were used for model training while the remaining 20% of data served as the test set. This process was repeated five times, with each fold serving exactly once as the test set, ensuring that every instance in the dataset contributed to both training and evaluation.

Performance metrics (accuracy, precision, recall, and F1-score) were calculated for each fold, and the mean and standard deviation across all five folds were computed to provide a comprehensive assessment of model stability and variance (Supplementary Fig. S1C). This approach mitigates the risk of performance estimates being dependent on a particular data split and provides confidence intervals for the reported metrics.

The stratified five-fold cross-validation served as a final validation step, confirming that the model’s performance generalizes consistently across different subsets of the data and is not an artifact of a particular train-test partition.

### FLYNC command line interface

FLYNC command line interface (CLI) is a powerful tool implemented in Python, built with cross-platform compatibility and ease of integration with existing bioinformatics tools and libraries in mind.

The CLI is designed to interact with a central shell script that orchestrates the execution of the pipeline. This script serves as the backbone of the FLYNC pipeline, managing the invocation of all child subprocesses and ensuring the sequential and conditional execution of various pipeline stages. The script provides visual cues from the pipeline, including progress updates for each main stage, allowing users to monitor the execution in real time. The program redirects the output of the child subprocesses to a central logging file “run.log,” enabling users to maintain a central record of the pipeline’s execution.

The CLI provides users with the ability to execute complex bioinformatics workflows through a series of simple, yet comprehensive commands. The FLYNC CLI offers multiple subcommands to accommodate various types of input data and user preferences: (i) “run”: Executes the full pipeline using a YAML configuration file, allowing for a high degree of customization with minimal command-line arguments, (ii) “sra”: Facilitates the analysis of SRA accession numbers by running the full pipeline on a list of provided accession numbers, (iii) “fastq”: Allows users to run the full pipeline on provided FASTQ files, supporting both paired and unpaired read inputs. Each subcommand is equipped with a set of options to specify input files, output directories, metadata for differential expression analysis, and the number of computational threads to utilize. The FLYNC CLI is invoked using the “flync” command followed by the desired subcommand and associated options. Examples of how to use the main subcommands: (i) To run the pipeline with a configuration file: “flync run -c /path/to/config.yaml,” (ii) To analyze a list of SRA accession number: “flync sra -l /path/to/list.txt -o /path/to/output/dir -m /path/to/metadata.csv -t 8,” (iii) To process local FASTQ files: “flync fastq -f /path/to/fastq/dir -o /path/to/output/dir -p true -m /path/to/metadata.csv -t 8.”

The “-c/--config” option is recommended as it simplifies the execution process by reading all necessary parameters from a YAML file. The “-l/--list” option is mandatory for the “sra” subcommand and requires a file with SRA accession numbers, one per line. The “-o/--output” directory is advisable for all subcommands to specify where the results will be written. Metadata in CSV format is mandatory for differential expression analysis and is specified with the “-m/--metadata” option. The number of computational threads can be adjusted using the “-t/--threads” option to optimize performance based on available system resources.

The FLYNC CLI is designed with user-friendliness in mind, providing clear and concise help messages for each subcommand. Running “flync < subcommand> --help” displays detailed information about the options available for that subcommand.

### FLYNC distribution and availability

FLYNC is distributed as a CLI tool to interact with the software pipeline and pretrained ML model that classifies lncRNAs. To ensure accessibility and ease of use across different computing environments, FLYNC is available through multiple distribution methods.

FLYNC is available as a Docker image, providing a containerized environment that ensures consistency across different platforms. The Docker image can be pulled from the Docker Hub repository (hub.docker.com/r/rfcdsantos/flync). Users with ample storage space may opt for the “local-tracks” tagged image, which is larger in size but offers faster runtime due to the inclusion of UCSC tracks for feature extraction. When using Docker, it is recommended to map a local directory to the container to store results. This ensures that the output is accessible on the host machine after the Docker process is completed.

For users operating on Debian-based Linux systems, FLYNC can be installed locally using Anaconda. This method allows users to test, modify, and inspect the FLYNC scripts directly. The repository can be cloned, and the required environments can be set up using the provided “conda-env” shell script.

FLYNC is distributed in a manner that prioritizes user convenience and reproducibility across different computing environments. The Docker and Conda distribution methods ensure that researchers can easily install and run FLYNC. FLYNC source code repository is hosted on GitHub (github.com/homemlab/flync) along with installation and usage instructions. Similarly, the SBUCELL module is available through GitHub (github.com/homemlab/subcell) with the same distribution approach.

### Bulk RNA-seq dataset

To assess the applicability of FLYNC, we utilized the publicly available bulk RNA-seq dataset GSE199164, which profiles gene expression in *D. melanogaster* brains at three post-eclosion ages: 3, 7, and 14 days. The dataset includes samples from both females and males, providing a view of sexually dimorphic gene expression in fruit fly brains under normal physiological conditions.

Because it spans multiple ages and sexes, this dataset provides a multifaceted biological context to test FLYNC’s adaptability across diverse experimental conditions. By applying FLYNC to this dataset, we can showcase the pipeline’s flexibility in handling complex experimental designs, including differential gene expression analysis across multiple biological variables. Moreover, this dataset offers a quick and reproduceable framework for experimental validation of FLYNC predictions by reverse transcription polymerase chain reaction (RT-PCR), given the ease of adult brain tissue collection.

### Single cell RNA-seq dataset

We also wanted to address the capacity of FLYNC to be applied to locally stored datasets. For that reason, we have resourced to scRNA-seq previously generated in our laboratory and already publicly available [26]. Also, considering the putative cell-type-specific function of lncRNAs, this dataset provides sequencing data for an array of neural populations, including neural stem cells (neuroblasts) and differentiated neural cells (neurons). The analysis was performed on reads pooled from 1418 Type I neuroblasts and 1791 neurons, which were identified and demultiplexed from the parent scRNA-seq dataset [26]. Hence, it would be possible to identify new putative lncRNAs, but also address its cellular context. Moreover, and as mentioned in the “Introduction” section, the brain, more than other organs already scrutinized, displays a tremendous amount and diversity of lncRNAs, thus supporting the reasoning behind the selection of this dataset. Finally, validation of these results could be easily verified by sorting pure populations of neuroblasts or neurons and testing via RT-PCR (see below).

### Fly husbandry

For experiments performed with whole heads, control (w1118), four 3-day-old male flies were collected. The whole head was extracted with forceps and immediately placed in ice-cold 250 μl 1× PBS. For the experiments performed with sorted cells, labeling of neural stem cells and their progeny was done by using the stable line expressing both VT201094-Gal4 (VDRC; central brain and ventral nerve cord neuroblast driver) and UAS-myr::GFP. To maximize expression of GFP, fly husbandry was set up at 29°C. After ~5 days, third instar wandering larvae were collected for brain dissection.

### Brain dissociation and cell sorting

Approximately two hundred wandering third instar larvae were collected and dissected in supplemented Schneider’s medium [10% fetal bovine serum (Sigma), 20 mM glutamine (Sigma), 0.04 mg/ml L-glutathione (Sigma), 0.02 mg/mL insulin (Sigma), and Schneider’s medium (Sigma)]. Afterwards, brains were enzymatically dissociated in Schneider’s medium supplemented with 1 mg/ml Papain (Sigma) and 1 mg/ml Collagenase I (Sigma) for 1 h at 30°C. Then, brains were washed twice with 1 ml supplemented Schneider’s medium. To help pellet the brains between washes, samples were spun at 300 × *g* for 5 min. Subsequently, brains were resuspended in 200 μl of supplemented Schneider’s medium and mechanically disrupted using a pipette tip. The cell suspension was filtered through a 30-μl mesh into a 5-ml FACS tube (BD Falcon) and immediately sorted by fluorescence-activated cell sorting (FACS) (FACS Aria III, BD). GFP-positive cells were collected in 750 μl of supplemented Schneider’s medium. Since neuroblasts (NBs) and their progeny can be distinguished by their size, NBs and neurons were collected separately [27]. Once cells were collected, additional PBS was added to reach a final volume of 1000 μl. Cells were then immediately used for RNA extraction.

### RNA extraction and RT-PCR

mRNA was isolated using TRIzol™ LS Reagent (Invitrogen) according to the manufacturer’s instructions. RNA was then treated with TURBO DNA-free™ Kit (Invitrogen™) to ensure removal of remnant DNA. cDNA was prepared using the RevertAid First Strand cDNA Synthesis Kit (Thermo Scientific™). RT-PCRs were done using GoTaq qPCR Master mix (Promega) on a QuantStudio™ 5 Real-Time PCR System (Applied Biosystems™). Expression of all genes was normalized to *act5C* or *vglut* and relative levels were calculated versus control using the ∆Ct method [28]. All measurements were done with technical triplicates. A list of the primers used can be found in Table 1.

**Table 1.** List of primers used for RT-qPCR experiments

Name	Sequence	Related to
act5C forward	GATAATGATGATGGTGTGCAGG	Figure 6A and C
act5C reverse	AGTGGTGGAAGTTTGGAGTG	Figure 6A and C
vglut forward	TGAGGTGCAATATGTCGGCG	Figure 6A
vglut reverse	TAGCCCCAGAAGAAGGACGA	Figure 6A
dpn intronic forward	CTCGACTGAAGTGGACTGCA	Figure 6A and C
dpn intronic reverse	GGGGCCCTACAACAAGTTCA	Figure 6A and C
MSTRG.19053 forward	AGTGGGAGTGTGTGTGTGTG	Figure 6A
MSTRG.19053 reverse	ACAGTAACCACCACCAGCAG	Figure 6A
MSTRG.6678 forward	ACACACACACTCATTCGGCT	Figure 6A
MSTRG.6678 reverse	CAGGGCAGAGCAACAACAAC	Figure 6A
MSTRG.8896 forward	GGCGGCTTCTTCCTCATACA	Figure 6A
MSTRG.8896 reverse	GCGACCTTGAACTCCTCGAA	Figure 6A
MSTRG.22805 forward	ATTACAACCACGGGCGACAT	Figure 6A
MSTRG.22805 reverse	CGCATTTGAGGCACACACTT	Figure 6A
MSTRG.3457 forward	GGCAATCACGATGGATGGGA	Figure 6A
MSTRG.3457 reverse	GATTGAGTGTGTGCCTGGGA	Figure 6A
MSTRG.13099 forward	CGATCGTGTGGACCCTTCAA	Figure 6A
MSTRG.13099 reverse	AGAAGCGGAAAGGAGAAGGC	Figure 6A
MSTRG.3880 forward	TCCCCGGCATTAATTCGCTT	Figure 6A
MSTRG.3880 reverse	CGATTGGAAAATGAGGGCGG	Figure 6A
MSTRG.4498 forward	GGCGAATTAGTTTTCCTTTGGC	Figure 6C
MSTRG.4498 reverse	TCGTGTCTGTGTGAGTGAGC	Figure 6C
MSTRG.4424 forward	AGCCAGGCCAAATCGAATCA	Figure 6C
MSTRG.4424 reverse	TGTATCTCGCTCGCTGGAAG	Figure 6C

## Results and discussion

### The *Drosophila melanogaster* noncoding genome annotation gap

**Figure 1.**
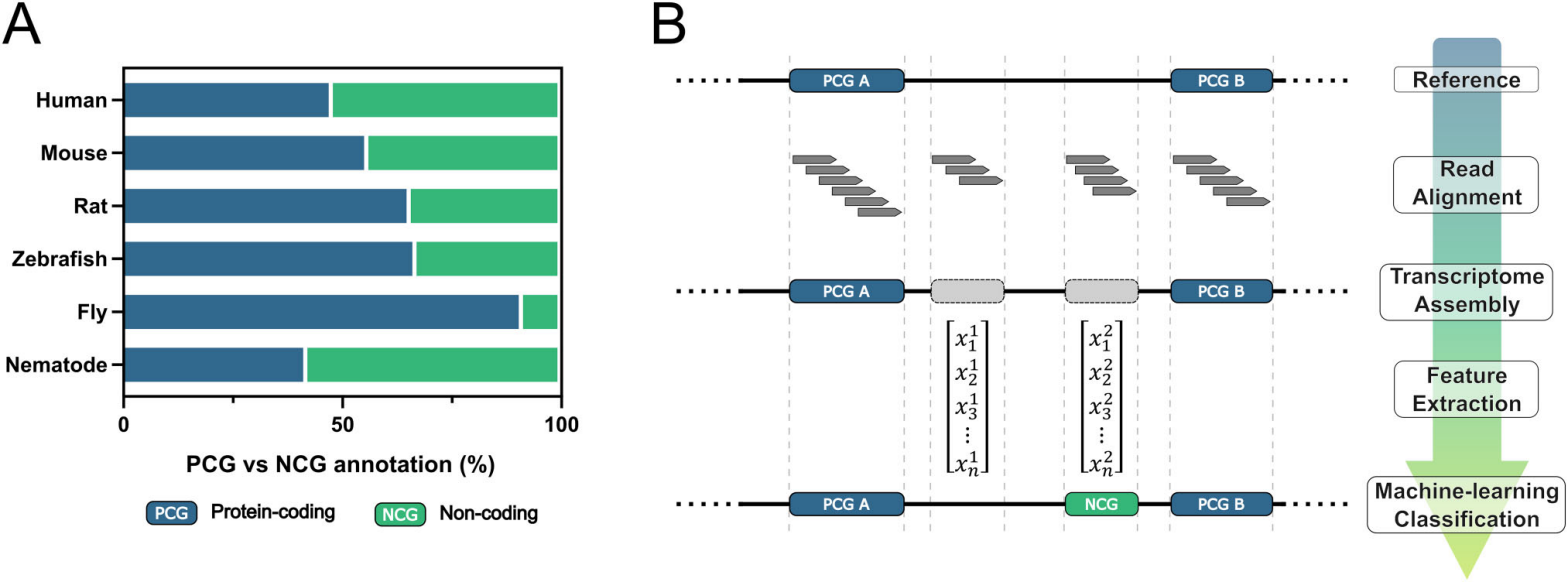
Bridging the annotation gap of ncRNAs in *D. melanogaster* with FLYNC. (**A**) Comparative analysis of Protein Coding Gene (PCG) and NCG, percent distribution in genome annotations across various model organisms. Analysis done using *D. melanogaster* reference genome BDGP6.46.113 r103. This figure illustrates the lower percentage of annotated NCGs in *D. melanogaster* compared to other species. (**B**) Schematic representation of the FLYNC ML approach for classifying RNA transcripts. The process includes building gene models from sequencing reads, constructing a feature matrix, and inferring classifications using a pretrained ML model.

*Drosophila melanogaster* is a well-established model organism for studying *in vivo* gene functions; however, lncRNAs in this species remain comparatively less well characterized [29]. To better understand this discrepancy, we compared the counts of PCGs and NCGs across various model organisms commonly used in research (Fig. 1A). Interestingly, organisms frequently employed in RNA biology research exhibit a higher percentage of annotated NCGs. For instance, in *Caenorhabditis elegans*, where RNAi was first discovered and characterized, NCGs constitute over half (55%) of the total annotated genes. In contrast, the proportion of annotated NCGs in *D. melanogaster* is significantly lower, at only 22% [30]. This underscores the potential presence of numerous undiscovered ncRNAs in *D. melanogaster*, possibly concealing a layer of RNA regulatory networks.

However, there is a lack of bioinformatics tools available for identifying NCGs specifically in *D. melanogaster*. To address this gap, we have developed FLYNC. FLYNC aims to bridge the annotation gap between the noncoding and coding genomes of *D. melanogaster* through an evidence-based gene discovery and ML-driven classification approach. The high-level overview of the FLYNC approach is the following: (i) building gene models from transcriptomic sequencing reads, (ii) creating an evidence-based feature matrix for each NCG model, and (iii) using a pretrained ML model to classify each gene as a previously unannotated RNA gene (Fig. 1B). This approach trains the ML model by feeding it with experimentally supported targets, which ultimately enriches the pool of true positive hits and helps validate candidate genes.

### Overview of FLYNC

**Figure 2.**
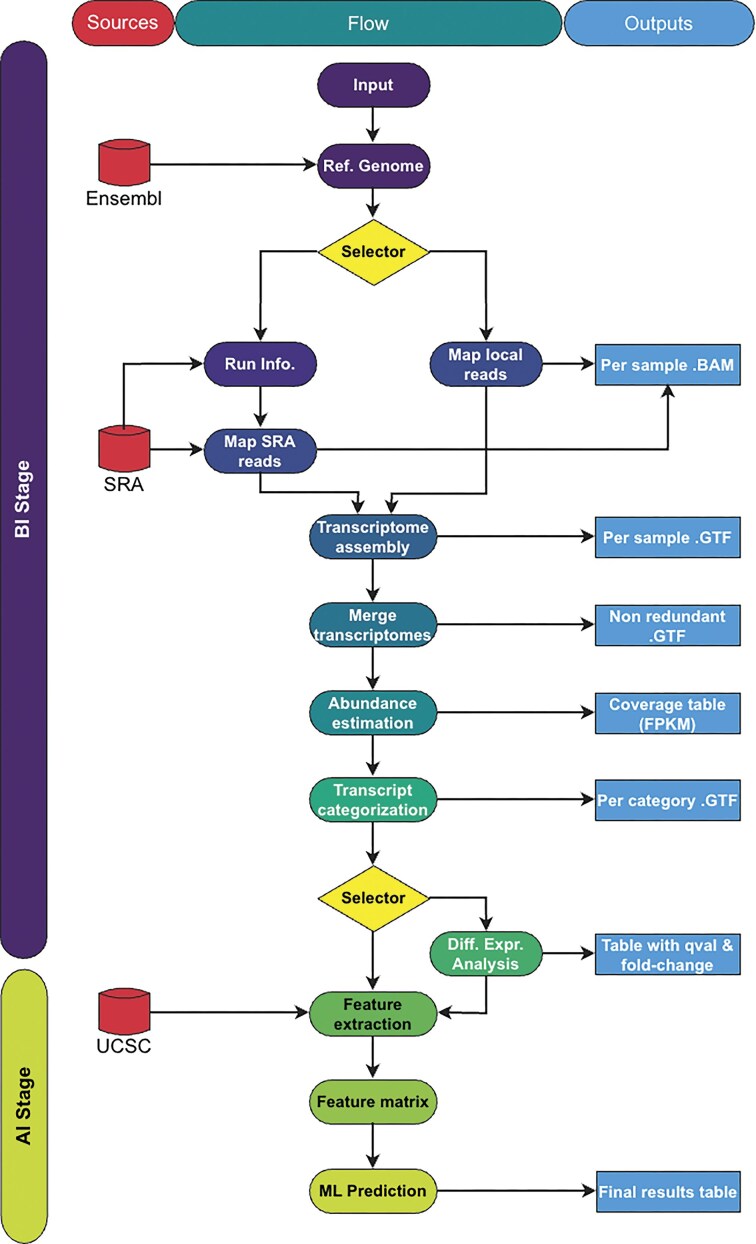
Overview of the FLYNC bioinformatics pipeline. The two main stages are depicted: BI and AI. The figure details the data flow from input through various analytical steps to the final output, highlighting the integration of external data sources (left) and the pipeline’s output components (right).

FLYNC was developed as an end-to-end pipeline to streamline its usage and simplify technical requirements for end users. FLYNC can be conceptually segregated into two main stages, a bioinformatics (BI) stage and an artificial intelligence (AI) inference stage (Fig. 2). The BI stage implements a bioinformatics pipeline based on HISAT2 [31] for alignment of RNA-seq reads to the genome and StringTie for reference-guided transcriptome assembly to generate transcripts, counts and merging assemblies It then uses Cuffcompare for comparing them against the reference annotation, GFFread for extracting the FASTA sequences of the newly identified, unannotated transcripts and Ballgown for downstream differential gene expression (DGE) analysis [32]. The AI stage refines the outputs of the BI stage querying the pretrained ML model to classify them as new lncRNAs.

The program depends on critical information from three different external public sources (Fig. 2—under “Sources”): Ensembl for the reference genome sequence and annotation; Sequence Read Archive for retrieving published transcriptomic reads; and the University of California Santa Cruz: Genomics Institute hosting the genome browser tracks (BigBed and BigWig files) used to build the features for the predictive model. During the computational steps, various files are created that may be used in future runs or fed into other bioinformatic pipelines (Fig. 2—under “Outputs”). The final “results_file.csv” comprises the summarized results for each new candidate lncRNA as classified by FLYNC. Namely, the probability of each new transcript being an lncRNA, genome coordinates, DEA information (if applicable) and per sample FPKM values.

The program will parse the user-given instructions (INPUT) to start the pipeline with the desired configuration. This requires: (i) the sequencing reads that will be mapped to the reference genome. These can either be “fastq” files supplied by the user or SRA accession numbers that will automatically trigger the download and local storage of the reads, (ii) the path to store the results of the pipeline. Optionally, a sample metadata file can be supplied to map each sequencing sample to a condition (e.g. wild type versus mutant). This can be used to run a DGE analysis on the newly assembled transcripts (Fig. 2—under “Flow”). Using this configuration, the program starts the pipeline by downloading the reference genome annotation and sequences to use at runtime (REF. GENOME). Based on the initial configuration, the program will either (SELECTOR): (i) map locally stored sequencing reads using the “fastq” subcommand (MAP LOCAL READS), (ii) map remotely stored sequencing reads retrieved from the SRA database along with vital SRA run metadata using the “sra” subcommand (RUN INFO. & MAP SRA READS). After all samples are mapped, the results are used to create an annotation of each sample’s transcriptome (TRANSCRIPTOME ASSEMBLY), which is then merged into a single non-redundant transcriptome annotation of all selected samples (MERGE TRANSCRIPTOMES). The quantity of each transcript is estimated (ABUNDANCE ESTIMATION) followed by a categorization of each gene (TRANSCRIPT CATEGORIZATION). Namely, into different subclasses of noncoding (new lincRNAs, intronic lncRNAs, antisense lncRNAs). To finalize the BI stage, the optional step (SELECTOR) of running a differential expression analysis (DIFF. EXPR. ANALYSIS) on the samples may be used to help refine the final output of the program. For each new candidate lncRNA transcript a set of features is pulled from the UCSC database (FEATURE EXTRACTION) and combined into a unified feature matrix of feature values per candidate lncRNA transcript (FEATURE MATRIX). Finally, we feed this matrix to our predictive model and store the result into a final dataset (ML PREDICTION).

### Training dataset and feature engineering

We reasoned that transcriptomic studies focused on different genomic and epigenomic properties, such as TF binding site distribution, histone marks or sequence conservation, and sequence-based properties, such as *k*-mer frequencies and RNA secondary structure, could be leveraged by AI algorithms to identify hidden patterns and help discover novel long noncoding transcripts. AI algorithms have been successfully used to identify lncRNAs for Human and Mouse organisms [33], but very few of these models have been tailored to specifically identify lncRNA genes in *D. melanogaster*. Moreover, the tools that provide species-agnostic capabilities often require high technical knowledge and/or a model retraining step.

To characterize the different genomic and epigenomic properties that can help identify lncRNAs we used several publicly available datasets. The UCSC Tracks Hubs provide curated, high-quality genomics tracks for *D. melanogaster*. We examined these data to identify possible features to train an ML model and included the relevant UCSC tracks in our pretraining dataset. This allowed us to: (i) engineer and evaluate features. (ii) refine feature selection for the final training dataset, (iii) determine the most suitable algorithm for model optimization.

**Figure 3.**
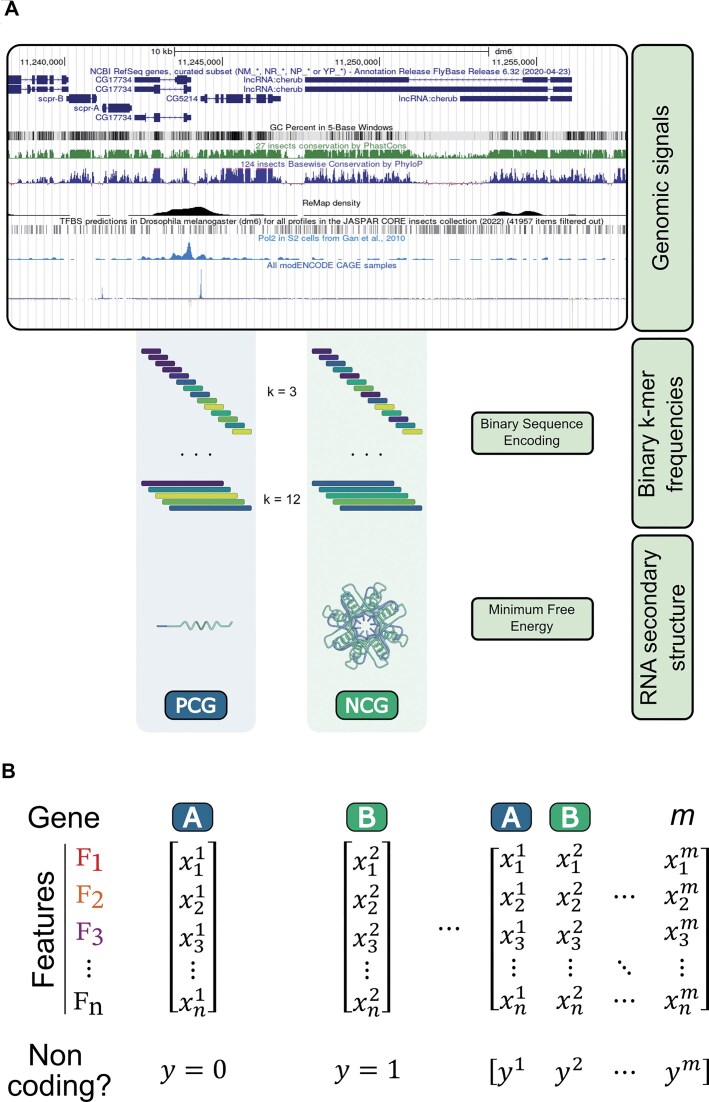
Building a gene feature matrix for FLYNC ML. (**A**) Visualization of the features used to train the FLYNC ML model, with an example genomic region displayed. Statistics of each track were used to extract various genomic and epigenomic features, such as transcription start sites or Transcription-factor binding. For sequence-level features, binary sequence encoding with *k*-mer frequency counting (3-mer to 12-mer) and minimum free energy (MFE) of RNA secondary structure were used to expand the feature set. (**B**) Schematic representation of the FLYNC inference feature matrix. Columns are genes defined by two genomic coordinates, while rows are the values of each feature. Each set of gene features has a corresponding binary value (PCG = 0; NCG = 1), which is assigned to the training dataset based on existing genome annotation, and the main output of the ML model during inference.

The groups of features used to train the AI model are ilustrated in Fig. 3A and Table 2, depicting an example of a genomic region to visually represent the source track data.

**Table 2.** Computational features used to train the lncRNA classification model

Feature Name(s)	Description	Count
mean_gc	The average GC content across the transcript.	1
cov_tfbs, max_tfbs	Coverage and maximum of a transcription factor binding site (TFBS).	2
cov_remap, cov_tfbs	Coverage and maximum of overlapping ReMap peaks.	1
cov_tss_plus, max_tss_plus, sum_tss_plus	Coverage, maximum, and sum of cap analysis of gene expression (CAGE) signal (plus strand).	3
cov_tss_minus, min_tss_minus, sum_tss_minus	Coverage, minimum, and sum of CAGE signal (minus strand).	3
cov_s2_pol2, sum_s2_pol2, max_s2_pol2	Coverage, sum, and maximum of RNA Polymerase II binding.	3
cov_h3k4me3, mean_h3k4me3, sum_h3k4me3	Coverage, mean, and sum of H3K4me3 histone modification.	3
cov_epdnew	The fraction of the upstream region covered by EPDnew promoters.	1
mean_pcons27, std_pcons27, sum_pcons27	Mean, standard deviation, and sum of PhastCons conservation scores (27 insect species).	3
mean_phylocons124, std_phylocons124, sum_phylocons124	Mean, standard deviation, and sum of PhyloP conservation scores (124 vertebrate species).	3
remap_*	A set of boolean features indicating overlap with ReMap ChIP-seq peaks.	549
tfbs_*	Boolean features indicating overlap with known JASPAR TFBS.	146
3mer_SVD1, …, 12mer_SVD1	The first principal component from SVD of TF-IDF weighted binary *k*-mer counts (for *k* = 3 to 12).	10
ss_mfe	The MFE of the RNA secondary structure.	1
Length	The length of the transcript.	1

For each set of features a short description is provided, as well as the number of features it contributes to the total number of 730. For ReMap and JASPAR extract features, “*” serves as a placeholder for the several protein transcription regulators present in this databases.

Our biologically driven feature engineering approach resulted in a total of 730 features designed to capture the multidimensional biological properties distinguishing lncRNAs from PCGs, encompassing regulatory, evolutionary, compositional, and structural dimensions (Supplementary Table S3). This integrated feature set combines data from multiple genomic sources and analytical strategies to form a robust classification framework (schematized in Fig. 3B).

The final feature set includes: (i) Quantitative genomic signals from various experimental datasets, (ii) Binary *k*-mer frequencies, (iii) MFE prediction of RNA secondary structure, (iv) A sparce matrix describing overlaps of TF binding sites and TF ChIP-seq signals. Together, these features capture complementary biological properties that enhance the model’s ability to discriminate between lncRNAs and PCGs. They can be grouped into three main categories:

- Genomic and epigenomic signals: These features reflect transcriptional and chromatin activity as well as evolutionary conservation. Examples include GC content, PhastCons and phyloP conservation scores, and regulatory protein binding coverage derived from ReMap and JASPAR datasets. In general, coding sequences show higher GC content and conservation than lncRNAs [34, 35]. The mean ReMap and JASPAR coverage features quantify the density of regulatory protein binding derived from large-scale DNA-binding datasets, positing that lncRNAs may demonstrate differing binding profiles relative to coding genes [36]. Similarly, coverage by RNA Polymerase II and H3K4me3 histone marks provides information on promoter activity and transcriptional engagement, which tend to be lower or more tissue-specific in lncRNAs. The inclusion of CAGE transcription start sites (TSS) features on both strands allows for the detection of distinct transcription initiation signatures, as lncRNAs often display broader or less defined TSS profiles compared to PCGs [37].
- Transcript-level annotations: TF binding information was integrated from two complementary databases: JASPAR and ReMap. This dual approach captures both sequence-based predictions and experimentally validated binding events. This approach generates a high-dimensional regulatory “fingerprint” for each transcript. The Boolean encoding (presence/absence for each TF) creates a sparse binary matrix that captures combinatorial regulatory logic and enables the model to identify characteristic regulatory signatures. Since lncRNAs and coding genes are often regulated by distinct TF networks, these features provide valuable insights into differential gene regulation.
- Sequence-based features: These capture compositional and structural properties, including GC content, *k*-mer profiles, MFE of RNA secondary structure, and transcript length. The *k*-mer profiles summarize sequence motifs that may reflect functional RNA elements such as binding sites for RNA-binding proteins. The MFE feature measures RNA secondary structure stability, a key determinant of lncRNA function and protein interaction potential. Additionally, sequence length provides another discriminative feature, as lncRNAs tend to exhibit less variability in length than coding transcripts [38].

The ReMap-derived features—which constitute the largest feature—are Boolean (binary) indicators of TF binding, where each feature represents the presence or absence of binding by a specific TF within the genomic region of interest, as determined by ChIP-seq experiments compiled in the ReMap database. While this creates a high-dimensional feature space, the sparse nature of these features results in a sparse matrix that reduces effective dimensionality, while maintining its direct biological interpretability.

To prepare the final training dataset a .GTF file of all *D. melanogaster* annotated genes (BFGP6.32 v103) was prepared by filtering out all tRNAs, rRNAs and sRNAs genes that excludes non-lncRNAs. This file was later used to extract the above features and compile them into a tabular dataset. To assign the target variable (label) for the ML model, all genes classified as “ncRNA” were assigned a value of True, while all others were assigned a value of False. The clear binary classification nature of the problem has the aim of accurately predicting whether a given transcript is a lncRNA or not. The complete training dataset is available in the Supplementary materials (Supplementary Table S2).

When selecting an appropriate AI programming methodology, we prioritized those that offered an optimal balance between performance and explainability. Consequently, we excluded unsupervised AI methods, which are often perceived as “black-boxes,” and instead focused on supervised and explainable ML frameworks [21]. To quickly test multiple ML algorithms, we prepared a pretraining dataset comprising a small, balanced sample of 10 000 randomly selected *D. melanogaster* NCG and PCG coordinates, from which we extracted the corresponding feature values. Using the “lazypredict” python library we conducted training session, reserving 15% of the original dataset to calculate multiple model metrics. Based on the initial algorithm screening, tree-based algorithms performed consistently well on the pretraining dataset. Results are provided in the Supplementary materials (Supplementary Table S1).

While many models, such as light gradient boosting machine or Xtreme Gradient Boosting (XGBoost) offer high-performance, they ultimately function as “black-boxes” and their lack of transparency can hinder biological interpretation [38]. We therefore focused on a class of models known as explainable AI (XAI), which are designed to be inherently interpretable. For this purpose, we selected the EBM as the core of our AI stage. EBMs are a type of generalized additive model that uses gradient boosting to train on one feature at a time. The result is a fully transparent model where the contribution of each feature to a final prediction can be precisely visualized and understood. This “glass-box” nature is highly advantageous for biological discovery, as it allows researchers to understand why a transcript is classified as a lncRNA by inspecting the learned feature graphs. Following the ML model development phase, we extracted the scores quantifying each feature’s contribution to the final model (Supplementary Fig. S1B). This analysis demonstrates the efficacy of the methodology and its utility for model interpretability. This inherent interpretability, combined with state-of-the-art performance, makes EBM uniquely suited for establishing an AI-powered bioinformatics framework to streamline *D. melanogaster* lncRNA discovery and study.

### Model training, evaluation and benchmarking

The EBM algorithm was selected as the optimal approach to achieve both high predictive performance and interpretability, allowing clear insight into how each feature contributes to classification outcomes. The EBM model was trained using the complete *D. melanogaster* genome. The training dataset exhibited a marked class imbalance, with 206 955 non-lncRNA (not-lncRNA) instances and 7708 lncRNA instances. The class imbalance in our training data reflects the annotation asymmetry between extensively characterized PCGs and the relatively understudied noncoding transcriptome that FLYNC aims to expand. We maintained this imbalance because: (i) it represents the realistic genomic composition encountered during novel lncRNA discovery, and (ii) evaluation during hyperparameter optimization revealed that resampling techniques—namely SMOTE and SMOTE-Tomek—not only failed to yield any significant improvement in overall model performance but actually reduced precision by 0.0215 and 0.0295 with SMOTE and SMOTE-Tomek, respectively, compared to directly fitting the imbalanced training set. This finding suggests that EBM’s ensemble architecture effectively handles class imbalance without requiring synthetic data augmentation [39].

**Figure 4.**
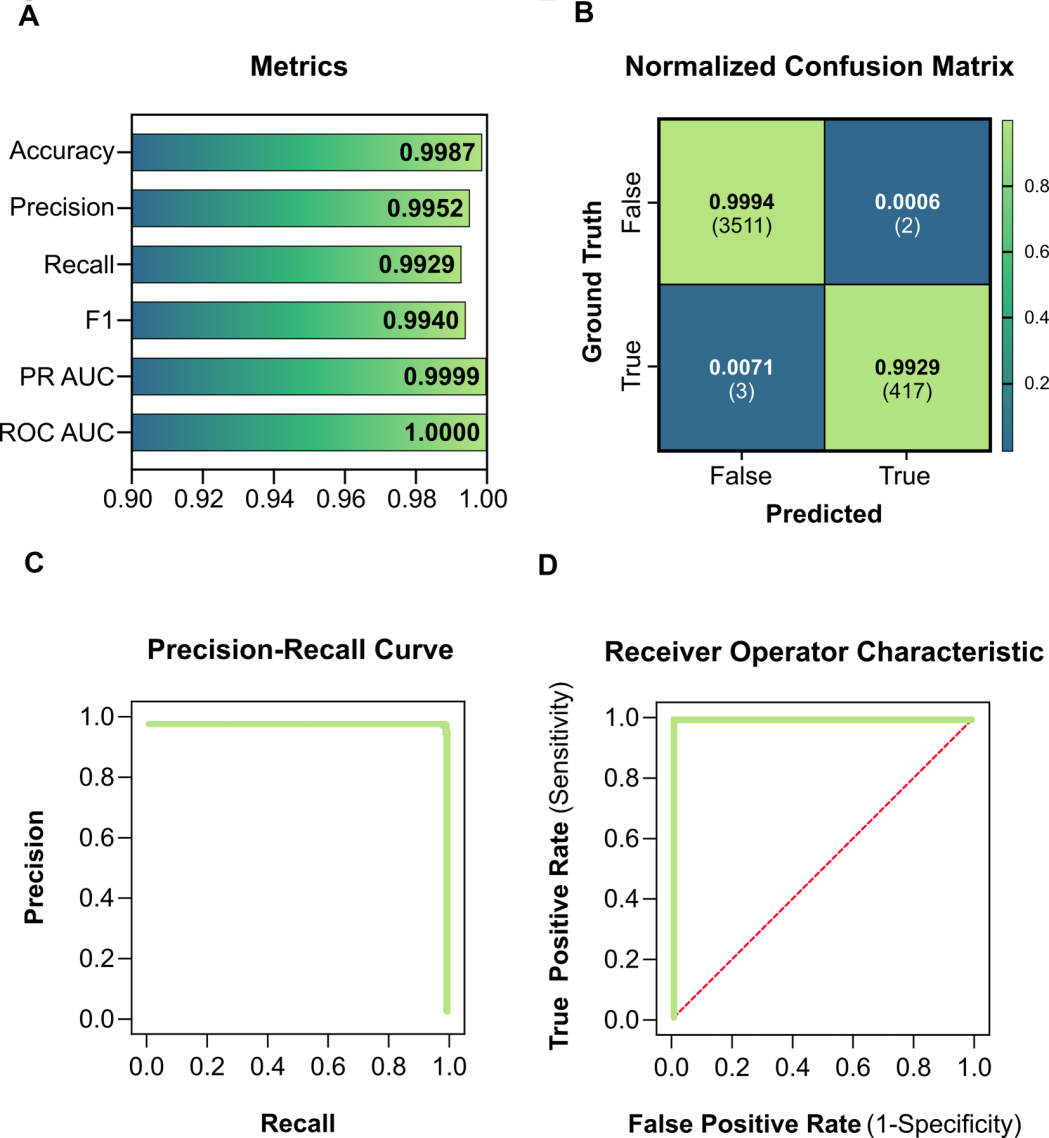
Evaluation of FLYNC EBM model on held-out data. (**A**) Graphical representation of the EBM model’s performance across four common metrics: accuracy, precision, recall, F1-score, PR AUC and ROC AUC with 0.9982, 0.9905, 0.9928, 0.9916, 0.9999 and 1.000, respectively. (**B**) Normalized confusion matrix for the RF model, showing the proportion of true and false predictions for non-lncRNA and lncRNA instances. Absolute instance counts of the hold-out dataset are shown between brackets. (**C**) Precision-recall curve for the EBM model, depicting the trade-off between precision and recall across different thresholds. (**D**) ROC curve for the EBM model, displaying the relationship between the TPR and FPR at various thresholds.

To assess the performance of the EBM model, we validated it using a hold-out dataset, with results summarized in Fig. 4A. The EBM achieved exceptional accuracy (0.9987), reflecting its ability to correctly classify the vast majority of instances. The precision score (0.9952) shows a very low rate of false positives, and the recall (0.9929) indicates that the model successfully identified almost all true lncRNAs. The F1-score (0.9940) confirms a strong balance between precision and recall. Additionally, the model attained near-perfect area under both the receiver operating characteristic (ROC) curve (ROC AUC: 1.0000) and the precision-recall (PR) curve (PR AUC: 0.9999), demonstrating its high discriminatory power even in the presence of significant class imbalance.

The normalized confusion matrix (Fig. 4B) further demonstrates that the model maintains high sensitivity and specificity across classes, irrespective of the class size disparity. Plots of the PR curve and ROC curve (Fig. 4C and D, respectively) further substantiate the model’s robust ability to distinguish between lncRNAs and other transcripts.

Importantly, because traditional metrics like accuracy and ROC AUC can be overly optimistic for imbalanced datasets—often reflecting majority class performance—the use of precision, recall, F1-score, and PR AUC is essential for a fair evaluation. The high PR area reinforces the model’s effectiveness in correctly identifying minority class instances (lncRNAs) and underscores its suitability for highly unbalanced genomic discovery tasks.

The EBM model performance metrics are at face value higher than those of widely used ML-based lncRNA identification models [40–42], and other lncRNA-related models [43, 44], which range 93.7%–99.1% accuracy. Even though, such metrics are often not easily comparable since some are calculated with a random balanced subsample of the training data or were tested on different species. To demonstrate FLYNC’s performance in a practical benchmarking context, we compared our model against LncDC, a lncRNA detection tool with demonstrated high performance in mammalian systems [23]. LncDC was originally optimized for human and mouse and offers the capability to retrain its ML model on new datasets, making it applicable to other organisms including *D. melanogaster*. For this comparison, LncDC was retrained on the same dataset used to train FLYNC, following the official guidelines and parameter recommendations provided by the LncDC development team. Our comparison therefore represents a realistic benchmarking scenario in which both tools are applied according to their respective best practices and documentation. To ensure fair evaluation, instances from FLYNC’s hold-out validation set were filtered out from the input files (CDS, cDNA, and ncRNA FASTA files from the same Ensembl release used to train FLYNC) provided to LncDC for retraining. The final evaluation and comparison with LncDC were performed on the identical hold-out set (15% validation split described in the “Materials and methods” section), providing a direct and unbiased measure of predictive performance (Supplementary Fig. S1A). Additionally, a comparison of computational resource utilization is provided in Supplementary Table S4, which highlights FLYNC implementation is substantially faster, with a much lower CPU demand, but higher memory footprint. Namely, FLYNC is 9.4× and 4.7× faster than LncDC for both training and inference workloads. This superior performance underscores the power of the features and model architecture used in FLYNC.

Furthermore, a key advantage of our new approach is its streamlined, end-to-end nature. The EBM can distinguish lncRNAs directly from all other transcripts, simplifying the workflow and removing potential sources of error from intermediate filtering steps. This makes FLYNC not only more accurate but also more efficient and user-friendly.

### Applying FLYNC to publicly available bulk transcriptomic data

We applied FLYNC to a publicly available bulk transcriptome sequencing dataset (GEO accession GSE199164) to investigate the presence of novel lncRNA in different groups of *D. melanogaster* samples. This sequencing data contains the transcriptome from 3, 7-days old male and female fly heads (brains) [45]. For this first analysis we selected six samples of 3-day-old Oregon wild-type flies, including three male and three female replicates. We provided their metadata to FLYNC for DGE analysis between male and female samples (Table 3).

**Table 3.** Phenotypic metadata for each SRA accession used in bulk RNA-seq analyses

Male versus female		3 days versus 7 days	
SRR18432803	Male	SRR18432803	3-days
SRR18432804	Male	SRR18432804	3-days
SRR18432805	Male	SRR18432805	3-days
SRR18432812	Female	SRR18432812	3-days
SRR18432813	Female	SRR18432813	3-days
SRR18432814	Female	SRR18432814	3-days
		SRR18432800	7-days
		SRR18432801	7-days
		SRR18432802	7-days
		SRR18432809	7-days
		SRR18432810	7-days
		SRR18432811	7-days

The table presents the metadata for two experimental setups: “male versus female” and “3 days versus 7 days,” reflecting the diverse conditions tested using the same transcriptomic dataset.

We used the CLI of FLYNC to run the workflow on these samples, as described in detail in the “Materials and methods” section. The command issued: “flync sra --list list.txt --metadata metadata.csv --output bulk_run.” This command instructed FLYNC to use the “sra” subcommand for SRA accessions, to read the sample list and metadata from the files “list.txt” and “metadata.csv,” respectively, and to store the results in a folder named “bulk_run.” The result for this run is provided as supplementary material files (Supplementary Table S5). Although no significative DGE were found in this analysis, it shows that this program is capable of performing DGE analysis that can then later be used to prioritize lncRNA functional studies.

FLYNC’s bioinformatics pipeline initially assembled a total of 60 049 transcripts across all 3-days whole heads samples after the transcript merging step. From this set, 20 759 transcripts were identified as potentially novel, meaning they were absent from the reference annotation. To distinguish novel from annotated transcripts, we filtered the Cuffcompare output GTF file based on transcript attributes: transcripts were classified as novel if they possessed a StringTie-generated transcript ID (e.g. a prefix “MSTRG.”) and lacked a ref_gene_id field linking them to an existing annotated gene. This novel set represents ~34.6% of the total assembled transcripts. Among these novel transcripts, the FLYNC ML classifier identified 128 as putative lncRNAs.

One of the advantages of FLYNC is its flexibility and adaptability to different experimental settings. For instance, we performed a different analysis using the same GEO dataset by grouping the samples by age instead of sex. Specifically, we compared the lncRNA expression profiles of 3-day-old (males and females combined) and 7-days old flies (males and females combined), to identify age-related lncRNA differences. This grouping by age, regartheless of sex, effectively increased the size of the dataset. We ran the FLYNC pipeline with the same command as before but updated the accessions in list.txt and changed the metadata.csv file to reflect the new grouping criteria (Table 3). In this second call, FLYNC identified a total of 66 309 transcripts, and from this 27 021 previously unannotated transcripts. FLYNC ML classified 193 as lncRNAs (Supplementary Table S6). Although no significant DEGs were identified in this analysis, this example demonstrates that FLYNC allows for straightforward comparisons across datasets, requiring only a simple configuration change and thereby simplifying the process for the end-user.

These analyses show how FLYNC can be easily applied to different biological questions and generate novel insights into lncRNAs, including the identification of sex-, tissue-, and stage-specific lncRNAs. Although no DGE was identified in this analysis, FLYNC’s pipeline offers this feature. The comprehensive understanding of lncRNA profiles and expression patterns is crucial for the unraveling of their functions and regulatory roles in biological systems. FLYNC is a powerful tool that offers flexibility and adaptability to different experimental settings and can easily incorporate data from different public transcriptomic datasets. For instance, additional datasets with 3- or 7-days old flies could be used to expand our analysis, and it would only require the user to add the required accessions and reflect the phenotypic data in the metadata.csv file. Remarkably, the FLYNC ML classifier resulted in a reduction of 99.994% (128 out of 20 759) and 99.993% (193 out of 27 021) in candidate lncRNAs for the first and second analyses, respectively. This significant reduction, based on the selection of highly probable true lncRNAs, will help streamline the discovery and characterization of novel lncRNAs in *D. melanogaster*. In conclusion, FLYNC is a valuable resource for the lncRNA research community and can facilitate the identification and analysis of lncRNAs in different conditions and contexts.

### Applying FLYNC to single-cell transcriptomic data

We next wanted to expand the applicability of FLYNC to single-cell RNA sequencing (scRNA-seq), one of the latest and most powerful sequencing methods. Importantly, this allows to withdraw cell-type specific information, something not possible from highly complex and heterogenous samples, as in the example of bulk-sequencing above. Typically, the output of the last step of the scRNA-seq pipeline is a matrix of gene expression values for each cell. This matrix can be used to identify different cell types and states, as well as to study gene expression patterns and regulatory networks [46]. The Seurat R package is one of the most popular and widely used scRNA-seq analysis tools [47]. However, the results are tied to the original annotated reference genome (for example, when using the 10× Chromium platform and the Cell Ranger software). Furthermore, we faced a challenge when trying to identify raw reads of each individual cell with the goal of using FLYNC. Since these reads are not easily accessible, we developed a performant program named “SUBCELL” (https://github.com/homemlab/subcell).

**Figure 5.**
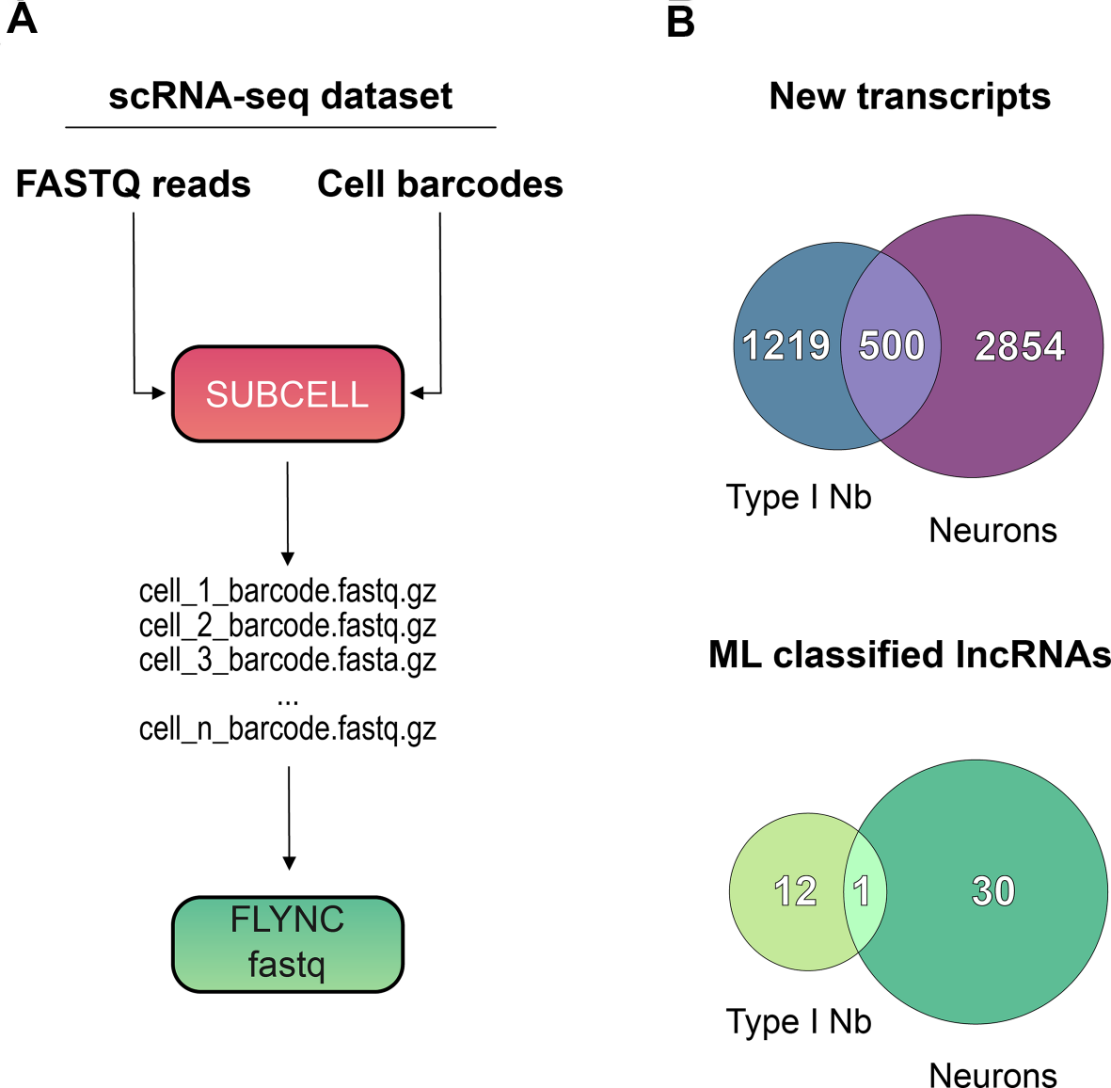
Applying FLYNC to single cell RNA-seq data with SUBCELL pre-processing step. (**A**) Diagram of the SUBCELL program’s functionality, which groups sequencing reads by cell barcode from the original FASTQ files, enabling their use in FLYNC ML pipeline for further analysis. (**B**) Summary of the FLYNC pipeline application to single-cell RNA-seq data for Type I Neuroblasts (Nb) and Neurons, showing the number of identified noncoding transcripts and the reduction in candidate lncRNAs achieved by the ML classifier.

SUBCELL takes as input the folder containing the original FASTQ files from the scRNA-seq experiment and the cell barcodes extracted using the “WhichCells()” function from the Seurat R package. It scans each FASTQ file to identify reads whose headers contain the specified cell barcodes and then groups all matching reads into new FASTQ files. This process effectively aggregates reads belonging to the same cell cluster as defined by the Seurat analysis. The resulting cluster-specific FASTQ files can then be used in downstream bioinformatics pipelines that require re-alignment or de novo assembly, such as the FLYNC pipeline (Fig. 5A).

To demonstrate this functionality, we extracted cell barcodes for Type I NB (*D. melanogaster* brain stem cells) and for neurons using the “WhichCells()” function, into two separate files: “neuroblasts.barcodes” and “neurons.barcodes.” We then ran SUBCELL with these barcode files to generate cell-type-specific FASTQ folders, each containing reads grouped by barcode. These datasets were subsequently processed using the FLYNC ML pipeline via the “fastq” subcommand.

A summary of the FLYNC results for Type I NBs and neurons is shown in Fig. 5B. The FLYNC bioinformatics pipeline initially assembled a total of 42 062 transcripts in NBs and 42 518 transcripts in neurons. From these sets, 2219 and 2832 transcripts were identified as potentially novel in NBs and in neurons, respectively. Of these novel transcripts, the FLYNC ML classifier further predicted 13 and 31 as putative lncRNAs in NBs and in neurons (Fig. 5B, Supplementary Table S7—lncRNAs predicted in single cell dataset from NBs, and Supplementary Table S8—lncRNAs predicted in single cell dataset from neurons). This effectively reduced the universe of candidate lncRNAs in 99.37% and 98.87% for NBs and neurons, respectively. Additionally, this comparative analysis identified several nonoverlapping candidate lncRNAs between the two cell types (Fig. 5B). These transcripts may represent cell-type-enriched lncRNAs, potentially involved in cell state regulation.

FLYNC maintained its ability to efficiently prioritize and filter the broad set of candidate lncRNAs, consistent with its performace in bulk transcriptomic analyses. Notably, this reduction in candidates is achieved through the ML classifier, which leverages features derived from genome-wide experimental datasets. This approach enhances the confidence in predicted positive hits and provides a reliable foundation for subsequent molecular characterization experiments.

### Experimental validation of FLYNC putative lncRNAs hits identified in the analysis of the bulk transcriptome datasets

We next sought to experimentally validate the performance of our pipeline and confirm that the identified lncRNAs correspond to genuine RNA transcripts rather than artifacts arising from genomic DNA or other contaminants. We have thus selected a subset of predicted lncRNAs for experimental testing. Since our bioinformatic analysis incorporated both bulk and single-cell RNA-seq datasets, we designed validation experiments specifically tailored to the characteristics of each dataset.

**Figure 6.**
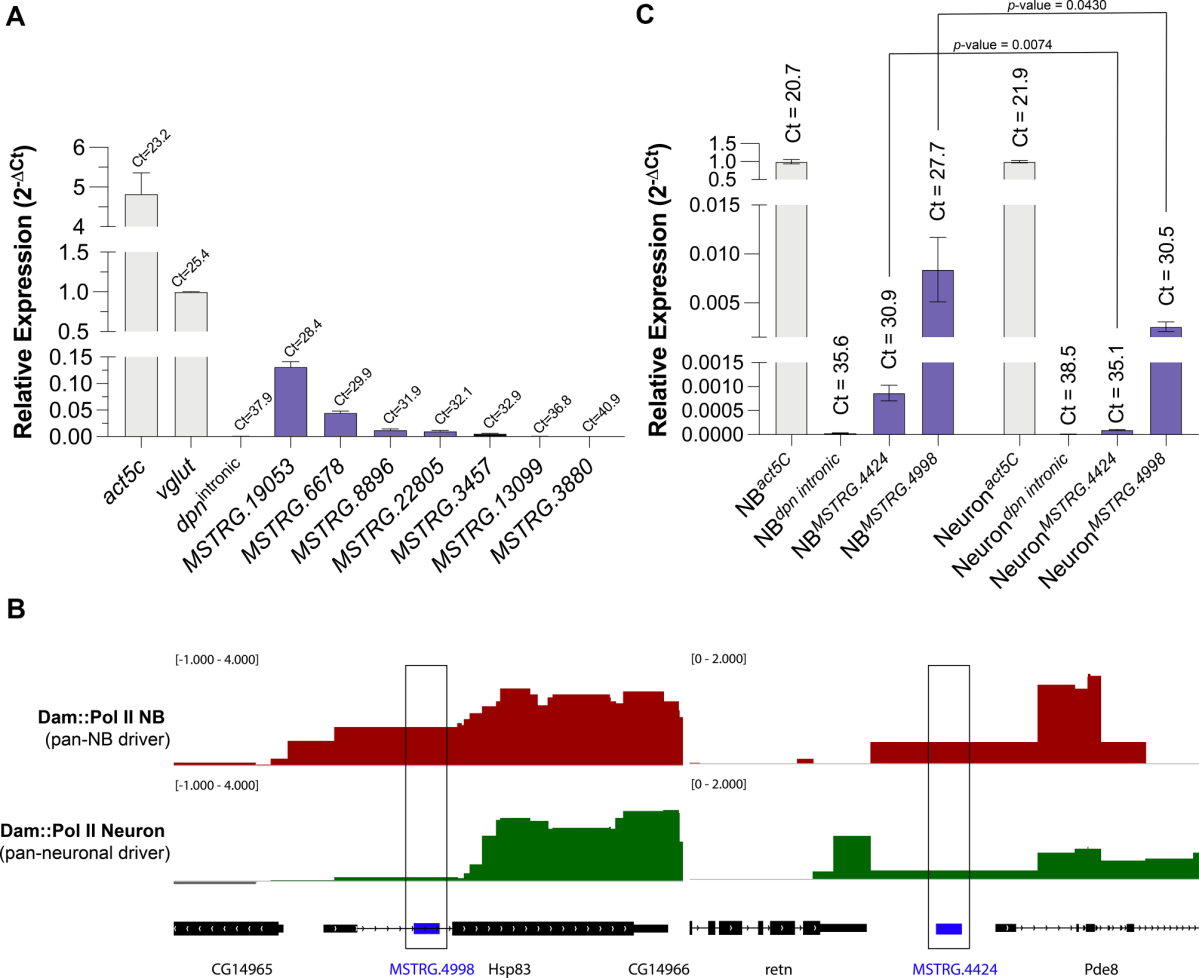
In vivo validation of FLYNC-identified lncRNAs. (**A**) RTqPCR validation of selected putative lncRNAs in RNA extracted from whole heads of 3-day-old adult males. Candidate lncRNAs were select based on their high predicted probability of being a true lncRNA by FLYNC and elevated expression levels. act5C and vglut were used as positive controls, while intronic dpn primers served as a negative control. Expression levels were normalized to vglut. Data represent mean ± standard deviation (SD) from three biological replicates and are shown as relative expression (2Δ-Ct). (**B**) Analysis of RNA Polymerase II (Pol II) recruitment at the loci of putative lncRNAs identified by FLYNC. Representative genome browser tracks from targeted DamID (TaDa) experiments showing RNA Polymerase II occupancy in neuroblasts (NB, red tracks) and neurons (green tracks) at two putative lncRNAs loci (MSTRG.4998 and MSTRG.4424). Expression of the Dam::PolII fusion protein was induced specifically in NBs or neurons using worniu (pan NB) and elav (pan neuronal) drivers, respectively. TaDa data are represented as log_2_ (Fold Enrichment) of Dam::Pol II over Dam-only (background correction). Genomic locations of NB-enriched putative lncRNAs identified by FLYNC are indicated with blue boxes, and the corresponding genomic regions are highlighted by a black box. (**C**) RTqPCR validation of the putative NB-enriched lncRNA indicated in panel (B), performed in RNA extracted from purified neuroblasts (NB) and neuron populations. Expression of the housekeeping gene act5C was used as positive control. Intronic dpn primers were used as negative control. Results are presented as relative expression (2Δ-Ct) normalized to act5C (mean ± standard deviation). Statistical analysis was performed to compare the relative expression of the selected lncRNAs in neuroblasts versus neurons (unpaired *t-*test, *n* = 3).

For the validation of the lncRNAs identified in the analysis of the bulk transcriptome datasets of adult heads, we have selected 7 putative lncRNAs to be experimentally validated by RT-PCR. First, RNA was extracted from 3-day-old adult male heads, followed by DNAse treatment to eliminate genomic DNA. The putative lncRNAs tested were selected based on two main criteria (i) a high FLYNC-predicted probability of being lncRNAs, (ii) relatively high expression levels (FPKM values -Fragments Per Kilobase of transcript per million mapped reads) within the dataset, to facilitate detection by reverse transcription polymerase chain reaction (RT-PCR) (Supplementary Table S6; primers used in Table 1). Expression of *act5C* and *vglut* genes served as positive controls, and results were normalized to the expression of the *vglut* gene. Both *act5C* and *vglut* are coding genes well expressed in this adult brain transcriptome dataset (GSE199164). As a negative control, primers targeting an intronic region of the *deadpan* (*dpn*) gene were used to exclude potential genomic DNA contamination and to define the threshold for non-expression. *dpn* encodes a TF specifically expressed in NBs and INPs, which are absent in adult stages; therefore, intron-containing pre-processed *dpn* transcripts are not expected in the adult fruit fly brain. The Ct value obtained for *dpn-*intronic amplification (Ct = 37.9) was used as the reference threshold for positivity. Accordingly, any amplification occurring before this threshold (Ct < 37.9) was considered indicative of expression, while any amplification beyond this value was classified as nonexpressed or nondetectable. Six out of the seven putative lncRNAs subjected to this analysis (MSTRG.19053, MSTRG.6678, MSTRG.8896, MSTRG.22805, MSTRG.3457, and MSTRG.13099) yielded RT-PCR products at a C_t_ lower than the defined threshold (Fig. 6A), confirming their expression. lncRNA MSTRG.3880 exhibited amplification beyond the defined threshold (C_t _= 40.9 versus C_t _= 37.9 for *dpn_intronic*). This likely reflects either the absence of transcription or expression levels below detection threshold. It is well established that several lncRNAs are expressed at very low levels [18]. Therefore, failure to detect some transcripts by RT-PCR does not preclude their existence or biological relevance. Lack of detection could also be due to technical factors such as low transcript stability or suboptimal primer efficiency. Additionally, while we sought to replicate the conditions of the analyzed datasets as closely as possible, the expression of some lncRNAs may be restricted to specific contexts (e.g. environmental stimuli) that were not replicated in our experimental setup. Finally, while the transcriptomic dataset used for model training was derived from adult brains, our experimental assays were conducted using whole heads, which may have diluted the abundance of these lncRNAs and reduced their detectability.

In sum, six out of seven tested putative lncRNAs were experimentally validated, without excluding the potential validity of the remaining candidates.

### Experimental validation of FLYNC-identified putative lncRNAs based on single cell transcriptomes

FLYNC identified several novel lncRNAs in NBs and neurons based on the analyses of scRNA-seq datasets. Because these lncRNAs were identified from cell type-specific datasets, they may be lowly represented when analyzed in the whole brain. To assess whether these *loci* are expressed, we first examined the recruitment of RNA Polymerase II (RNA Pol II) to their genomic *loci* using Targeted DamID (TaDa) (Fig. 6B) [48]. Briefly, TaDa allows profiling genome-wide recruitment of a given protein. Specifically, an RNA Pol II subunit was fused to the bacterial adenine methyltransferase (Dam), which methylates adenines within GATC sequences near RNA PolII binding sites [49]. Sequencing of these methylated *loci* serves as a proxy for PolII occupancy. By expressing Dam::RNA Pol II under cell type specific drivers, *worniu*-GAL4 for NBs and *elav*-GAL4 for neurons, TaDa generates high-resolution, cell-type-specific transcriptional profiles.

To streamline this analysis, we focused on the lncRNAs identified in the NB dataset, aiming to validate their existence by an independent method and to assess their enrichment in NBs. There were 13 putative novel lncRNAs specificaly identified in the NB population dataset. Representative genome browser tracks of RNA Pol II recruitment for two of these lncRNAs (MSTRG.4998 and MSTRG.4424) are presented in Fig. 6B. Both *loci* display prominent RNA Pol II peaks in NBs (red tracks), consistent with active transcription, while signal is markedly reduced in neurons (green tracks). These results support the transcriptional activity of these loci and their enrichment in the NB population.

To further validate these findings, we performed RT-PCR on RNA extracted from purified populations of NBs and neurons. Cells were isolated by FACS using a neuroblast-specific Vienna Tile GAL4 line (#201094) to drive expression of membrane-bound CD8::GFP in central brain and ventral nerve cord NBs [26]. Because GFP is stable and inherited by progeny, both NBs and their neuronal descendants were efficiently labeled [26]. Sorting based on fluorescence intensity and cell size allowed clear separation of NBs from neurons [27, 50]. Total RNA was extracted, treated with DNase to remove genomic DNA, and analyzed by RT-qPCR using intronic *dpn* primers as a negative control and *act5C* as a housekeeping positive control (expressed both in NBs and neurons). We have selected two candidate lncRNA genes (MSTRG.4424 and MSTRG.4998) displayed in Fig. 6B for further analyses. As depicted in Fig. 6C, it was possible to observe amplification of these lncRNAs in a pure population of NBs (left side of the graph), but to a lesser extent in neurons (right side of the graph). Quantitatively, MSTRG.4424 expression was significantly enriched by 8.6-fold in NBs compared to neurons (8.67 × 10^−4^ versus 1.01 × 10^−4^, respectively, *P*-value = 0.074). MSTRG.4998 was significantly enriched by 3.3-fold in NBs relative to neurons (8.40 × 10^−3^ in NBs versus 2.56 × 10^−3^ in neurons, *P *= 0.043). This experiment confirms the NB-specifc enrichement of these newly identified lncRNAs.

Our analyses validate the predictive accuracy of FLYNC and demonstrate that it is a powerful and reliable tool for identifying lncRNAs in *D. melanogaster*. The combined *in silico* and *in vivo* evidence underscores the robustness of the pipeline, successfully identifying previously unknown lncRNAs within a diverse array of pre-existing datasets. This not only reinforces the accuracy and utility of FLYNC but also opens up new avenues for understanding the complex regulatory roles of lncRNAs in *D. melanogaster*.

## Conclusion

In this study, we have addressed a significant gap in the annotation of the noncoding genome of *D. melanogaster*, a model organism paramount to the understanding of highly conserved developmental pathways and human disease mechanisms. Despite its widespread use, the *D. melanogaster* noncoding genome has remained largely underexplored, particularly in comparison to other model organisms. Our findings underscore the potential for a vast array of ncRNAs yet to be discovered, which may play crucial roles in RNA regulatory networks. To bridge this gap, we developed FLYNC, an innovative bioinformatics pipeline that leverages an ML-driven classification approach to identify and annotate NCGs. FLYNC integrates a robust BI stage, based on established protocols and coding potential assessment, with an AI inference stage that refines and classifies the outputs as new lncRNAs. The pipeline effectively utilizes data from Ensembl, the Sequence Read Archive, and UCSC Genomics Institute, ensuring a comprehensive and evidence-based approach to gene discovery.

The EBM model, central to the AI stage of FLYNC, showed exceptional performance with near-perfect accuracy and precision. Its performance surpasses other existing lncRNA identification models [23, 33, 44], and its inherent transparency and interpretability will facilitate its adoption and refinement by the research community.

Our results demonstrate that FLYNC is not only capable of identifying a significant number of previously unannotated transcripts but also classifies these with high precision and reliability. The application of FLYNC to both bulk and single-cell transcriptomic data illustrates its versatility and effectiveness in discovering novel lncRNAs across different biological contexts and experimental settings. FLYNC’s utility was further evidenced by its application to publicly available transcriptomic datasets, where it successfully identified and classified lncRNAs, providing a foundation for future functional and regulatory studies. Moreover, the integration of SUBCELL with FLYNC extended the pipeline’s applicability to single-cell RNA-seq data, highlighting the potential for cell-type-specific lncRNA discovery. Notably, SUBCELL can also be applied to 10× Genomics generated scRNA-seq datasets from various organisms, making it a valuable tool for researchers looking to re-analyze genomes and investigate cell-type-specific expression of novel genes, isoforms, and more.

In summary, FLYNC represents a significant advancement in the study of the noncoding genome of *D. melanogaster*. It is a powerful and adaptable tool that streamlines the discovery of lncRNAs, enabling researchers to uncover the hidden layers of RNA regulation. As such, FLYNC is a valuable addition to the toolkit of geneticists and molecular biologists, promising to enhance our understanding of the complex regulatory networks that underpin biological function and disease.

## Supplementary Material

lqaf216_Supplemental_Files

## Data Availability

FLYNC is an open source software available through an GPL-3.0 license in the GitHub repository (github.com/homemlab/flync) and in Zenodo by the permanent DOI https://doi.org/10.5281/zenodo.17878810. SUBCELL is an open source software available through an GPL-3.0 license in the GitHub repository (github.com/homemlab/subcell) and in Zenodo by the permanent DOI https://doi.org/10.5281/zenodo.17878266. Bulk RNA sequencing can be found in the Gene Expression Omnibus website (GSE199164; https://www.ncbi.nlm.nih.gov/geo/query/acc.cgi?acc=GSE199164). Single-cell RNA sequencing data can be found in the Gene Expression Omnibus website (GSE179763; https://www.ncbi.nlm.nih.gov/geo/query/acc.cgi?acc=GSE179763). Published and newly generated Targeted DamID data can be found in the Gene Expression Omnibus website (GSE77860, https://www.ncbi.nlm.nih.gov/geo/query/acc.cgi?acc=GSE77860; GSE282899, https://www.ncbi.nlm.nih.gov/geo/query/acc.cgi?acc=GSE282899).
