# Supplementary material for: FLYNC: a machine-learning-driven framework for discovering long noncoding RNAs in Drosophila melanogaster
Source: NAR Genom Bioinform. 2026 Jan 15;8(1):lqaf216. doi: 10.1093/nargab/lqaf216 (PMC12805895; doi:10.1093/nargab/lqaf216)
Supplement: lqaf216_Supplemental_Files [file lqaf216_supplemental_files.zip › Supplementary Material_R2_Clean.pdf]

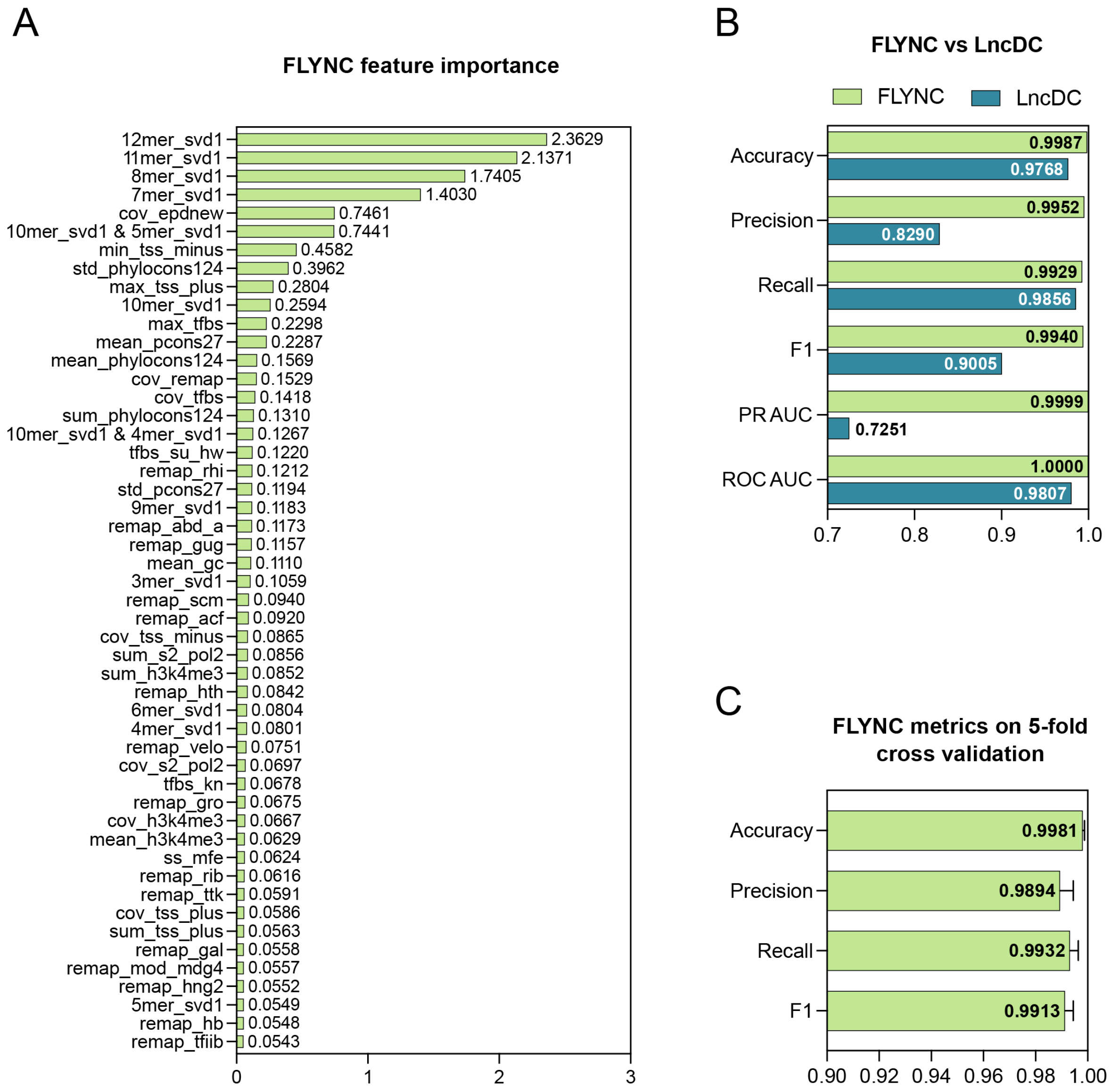

Supplementary Figures

Supplementary Figure 1 – FLYNC performance metrics and comparison with LncDC. **(A)** Feature importance rankings showing the relative contribution of each feature to the predictive output of the FLYNC model. **(B)** Comparison of performance metrics (accuracy, precision, recall, F1-score, PR AUC, and ROC AUC) between FLYNC (green) and LncDC (blue) evaluated on an identical hold-out dataset. **(C)** Cross-validation performance of FLYNC, showing accuracy, precision, recall, and F1-score for each fold; mean and standard deviation across five folds illustrate model stability and variance.

Supplementary Tables

Supplementary Table 1 – Performance Comparison of Machine Learning Classifiers. This table summarizes the performance metrics of various machine learning classification models evaluated on the same dataset. Each model was assessed using multiple performance indicators, including accuracy, balanced accuracy, ROC AUC, F1 score, precision, and time taken for model training.

Supplementary Table 2 – Table containing the complete training dataset used in this manuscript.

Supplementary Table 3 – Importance scores of each feature in determining the predictive output of our model pipeline.

Supplementary Table 4 - Comparison of computational resourse utilization in FLYNC *versus* LncDC.

Supplementary Table 5 – LncRNAs identified by FLYNC in datasets from whole brain bulk RNA sequencing in 3-days old male flies *versus* 3-days old female flies. Table summarizing transcript genomic location (chromosome and start/end sites), sequence, predicted lncRNA probability and classification (TRUE or FALSE), and RNA expression level across datasets, including average expression (FPKM).

Supplementary Table 6 – LncRNAs identified by FLYNC in datasets from whole brain bulk RNA sequencing in 3-days old male&female flies *versus* 7-days old male&female flies. Table summarizing transcript genomic location (chromosome and start/end sites), sequence, predicted lncRNA probability and classification (TRUE or FALSE), and RNA expression levels across datasets, including average expression (FPKM).

Supplementary Table 7 - LncRNAs identified by FLYNC in datasets from single-cell RNA sequencing in neuroblasts. Table summarizing transcript genomic locations (chromosome and start/end sites), sequences, and predicted lncRNA probabilities and classifications (TRUE or FALSE).

Supplementary Table 8 - LncRNAs identified by FLYNC in datasets from single-cell RNA sequencing in neurons. Table summarizing transcript genomic locations (chromosome and start/end sites), sequences, and predicted lncRNA probabilities and classifications (TRUE or FALSE).
